# Anti-Inflammatory Activity and Mechanism of Hydrostatin-SN1 From *Hydrophis cyanocinctus* in Interleukin-10 Knockout Mice

**DOI:** 10.3389/fphar.2020.00930

**Published:** 2020-06-19

**Authors:** Chuan Zhang, Shanshan Guo, Junjie Wang, An Li, Kuo Sun, Lei Qiu, Jianzhong Li, Sheng Wang, Xingyuan Ma, Yiming Lu

**Affiliations:** ^1^State Key Laboratory of Bioreactor Engineering, East China University of Science and Technology, Shanghai, China; ^2^School of Medicine, Shanghai University, Shanghai, China; ^3^Department of Critical Care Medicine, Shanghai Tenth People’s Hospital, Tongji University School of Medicine, Shanghai, China; ^4^Department of Biochemical Pharmacy, School of Pharmacy, Second Military Medical University, Shanghai, China; ^5^Department of Critical Care Medicine, Shanghai Tenth People's Hospital, Tongji University, Shanghai, China

**Keywords:** Hydrostatin-SN1, type I TNF-α receptor, anti-inflammation, colitis, *Hydrophis cyanocinctus*

## Abstract

Biopeptides derived from marine species have garnered significant research interest owing to their anti-inflammatory, antibacterial, and anticancer activities. In our previous study, Hydrostatin-SN1, a bioactive peptide extracted from the *Hydrophis cyanocinctus* venom gland T7 phage display library, demonstrated anti-inflammatory activity in a dextran sulfate sodium-induced murine colitis model. In this study, we investigated the anti-inflammatory activity and the underlying mechanism of Hydrostatin-SN1 in lipopolysaccharide (LPS)-induced bone marrow-derived macrophage (BMDM) cells and interleukin (IL)-10 knockout mice. The results showed that Hydrostatin-SN1 inhibited phosphorylation of JNK, ERK1/2, and p38 and decreased the mRNA expression of tumor necrosis factor-α (TNF-α), IL-6, and IL-1β in LPS-stimulated BMDM cells in a dose-dependent manner. In LPS-induced acute shock model, a significant higher survival rate of Hydrostatin-SN1-treated mice was observed. Furthermore, Hydrostatin-SN1 reduced body weight loss, decreased disease activity index, reduced spleen index, prevented histological injury, and inhibited the expression of IL-β and phosphorylation of JNK, ERK1/2, and p38 in the colon tissue of IL-10 knockout mice. Additionally, the positive expression rate of TNF-α in mice colon was decreased. Overall, our results suggest that Hydrostatin-SN1 has significant anti-inflammatory effects, both *in vitro* and *in vivo*.

## Introduction

Tumor necrosis factor-α (TNF-α) is a major pro-inflammatory cytokine, mainly expressed by activated macrophages and T lymphocytes, which plays an important role in regulating pro-inflammatory responses and biological processes (Aggarwal et al., 2012). Soluble TNF-α binds to two typical receptors, type I TNF-α receptor (TNFR1; also known as TNFRSF1A, CD120a, p55) and type II TNF-α receptor (TNFR2; also known as TNFRSF1B, CD120b, p75). The structures and expression patterns of these two receptors are completely different (Braumüller et al., 2013; Mehta et al., 2018). While TNFR1 is expressed in almost all mammalian cells, the expression of TNFR2 is mainly restricted to immune cells and endothelial cells, and it is responsible for the regulation of cell survival and repair (Al-Lamki et al., 2009; Faustman and Davis, 2010). The binding of TNF to TNFR1 mediates the activation of downstream inflammatory signal pathways (Ashkenazi and Dixit, 1998; Ting and Bertrand, 2016). This process is associated with a variety of autoimmune diseases, such as rheumatoid arthritis (RA) and inflammatory bowel disease (IBD) (Ashkenazi and Dixit, 1998; Wajant and Scheurich, 2011; Brenner et al., 2015). The anti-TNF-α agents, currently used in clinical treatment, are unable to directly or specifically target TNFR1, which results in various severe side effects (Sicotte and Voskuhl, 2001; Brown et al., 2002; Shakoor et al., 2002; Sedger and McDermott, 2014). Despite the discovery of the crystal structure of TNFR1 over the past years, reliable and potent TNFR1 inhibitors have not yet been developed (Mukai et al., 2009; Nair and Wilson, 2019). Thus, therapeutic targeting of TNFR1 signaling is a promising approach. Thus, there is a need to identify novel agents that can target TNFR1.

Over the past decades, researchers have committed to identify potential sources of new bioactive natural products from marine species (Wei et al., 2015). Peptides are important biologically active natural products found in several marine species (Zhou et al., 2018). In-depth studies on the biological functions and action mechanisms of these marine bioactive peptides have been conducted to identify potential drugs for the treatment of cancer (Rinehart et al., 1981; Rambow et al., 2018), diabetes (Admassu et al., 2018), and hypertension (Gogineni and Hamann, 2018). Currently, bioactive peptides from marine species are widely used in different areas of drugs development owing to their antibacterial, antiviral, anticancer, hypoglycemic, and cytotoxic properties (Sable et al., 2017). Animal venom is rich in peptides and other components, and can be modified to alter the physiology of other organisms effectively, rapidly, and selectively. It is a natural source of potential drugs, and has received increased attention. Therefore, we believe that the toxins obtained from marine organisms also have a great potential for drug development.

In our earlier research, a 22-amino acid peptide (Hydrostatin-SN1) was identified and was shown to bind to TNFR1 and exert its anti-inflammatory effect by inhibiting the MAPK and NF-κB pathways both *in vitro* (Zheng Y. et al., 2016) and *in vivo* (Wu et al., 2017). In the present study, we further explored the anti-inflammatory effect of Hydrostatin-SN1 *in vitro* and *in vivo*, using corresponding models. We believe that these results will provide a theoretical basis for the development of Hydrostatin-SN1 as a novel TNFR1 antagonist peptide.

## Materials and Methods

### Biological Materials and Reagents

Hydrostatin-SN1 and a random peptide were synthesized by ChinaPeptides Co., Ltd (Shanghai, China). Macrophage-colony stimulating factor (M-CSF) was bought from PeproTech Inc. (Rocky Hill, NJ, USA). RMPI 1640 medium, penicillin, and streptomycin were purchased from Hyclone Laboratories, Inc. (Logan Cache, UT, USA). Fetal bovine serum (FBS) was purchased from Thermo Fisher Scientific Inc. (Waltham, MA, USA). *Escherichia coli* 055: B5 LPS L2880 and piroxicam were purchased from Sigma–Aldrich Chemical Co. (St. Louis, MO, USA). Infliximab was bought from Remicade Company. TRIzol agent and SYBR Green PCR Master Mix were purchased from Takara Biomedical Technology (Beijing) Co., Ltd. (Beijing, China). JNK, phospho-JNK, ERK1/2, phospho-ERK1/2, p38, phospho-p38, and glyceraldehyde-3-phosphate dehydrogenase (GAPDH) monoclonal antibodies were purchased from Cell Signaling Technology Inc. (Beverly, MA, USA). Other chemicals and reagents used in this study were of analytical grade.

### Animals

C57BL/6 male mice (20–25 g) were purchased from the Experimental Animal Center, Second Military Medical University (Shanghai, China). IL-10-knockout (KO) mice (20–25 g, 6–8 weeks old) were purchased from Cavens Lab Animal Ltd. (Changzhou, China). The mice were housed in individual cages under controlled conditions (25°C, 50% humidity, and 12 h day/night cycle), with free access to food and water. All animal experiments were conducted according to the Guide for the Care and Use of Laboratory Animals published by the National Institutes of Health, and the study protocol was approved by the Animal Care and Use Committee of the Second Military Medical University.

### Bone Marrow Cell Culture

Bone marrow cells, extracted from the 4–6 weeks old male C57BL/6 mice, were suspended in RMPI 1640 medium (containing penicillin, streptomycin, and 10% FBS (Weischenfeldt and Porse, 2008) and cultured in 6-well flat bottom plates (3 × 10^7^ cells/well) for 3 days (37 °C, 5% CO_2_) in the presence of M-CSF (20 ng/mL). The medium was replaced with fresh medium containing M-CSF (20 ng/mL) on the third day. Six days later, in the positive group were treated with 800 µg/mL Infliximab, while the cells in other groups were treated with different concentrations of Hydrostatin-SN1 (10, 20, 40, and 80 μM) for 30 min, followed by incubation with or without 1 μg/mL LPS for 6 h.

### Quantitative Real-Time PCR

The total RNA was extracted from cells and colon tissues using TRIzol agent. Quantitative real-time PCR (RT-PCR) was carried out using the SYBR Green PCR Master Mix with the reaction mixture of total volume 10 μL on the Step two Plus Real-Time PCR System (Applied Biosystems). The sequences of the primers used were as follows: *GAPDH* (FP: AGG TCG GTG TGA ACG GAT TTG; RP: TGT AGA CCA TGT AGT TGA GGT CA), *IL-6* (FP: CCA ATG CTC TCC TAA CAG AT; RP: TGT CCA CAA ACT GAT ATG CT), *IL-1β* (FP: TTC AGG CAG GCA GTA TCA; RP: GTC ACA CAC CAG CAG GTT AT), and *TNF-α* (FP: TGA ACT TCG GGG TGA TCG GTC; RP: AGC CTT GTC CCT TGA AGA GGA C).

### Western Blot

The cells and colon tissues were lysed with lysis buffer after washing with ice-cold PBS buffer. The extract was centrifuged (4°C, 5,000 rpm, 10 min) and the supernatant was collected. Protein concentration was determined using the BCA protein assay kit. Western blot was performed with JNK, phospho-JNK, ERK1/2, phospho-ERK1/2, p38, phospho-p38, and GAPDH monoclonal antibodies. Relative protein levels were quantified using Quantity One software and expressed as optical density ratio.

### Model of Acute Shock Induced by LPS

C57BL/6 male mice (6–8 weeks old) were randomly divided into three groups (n = 8, each): one control group and two Hydrostatin-SN1 groups (50 and 250 μg/kg). LPS-induced inflammation model of mice was established, as previously described (Steeland et al., 2018). Briefly, all experimental mice were injected with 15 mg/kg LPS. Then, the mice in the Hydrostatin-SN1 groups were injected with the corresponding dosage of Hydrostatin-SN1, while those in the control group were injected with an equal volume of saline. The survival rate of the mice in each group was observed every 6 h, and the corresponding survival curves were plotted.

### Spontaneous Colitis in IL-10 KO Model

Male IL-10-KO mice were administered 10 mg/kg/d piroxicam by oral gavage. The mice that presented with inflammatory symptoms (diarrhea, hematochezia, severe weight loss, rectal prolapsed, and other symptoms), were then randomly divided into the following four groups: (1) IL-10 KO group (n = 8), the mice were administered 100 μL of saline by intraperitoneal injection each day; (2) IL-10 KO + Hydrostatin-SN1 group (n = 8), the mice were administered 50 μg/kg Hydrostatin-SN1 by intraperitoneal injection each day; (3) IL-10 KO + IFX group (n = 8), the mice were administered 4 mg/kg/d infliximab by intraperitoneal injection each day; (4) IL-10 KO + RP group (n = 8), the mice were administered 800 μg/kg/d random peptide by intraperitoneal injection each day. The day on which the mice were successfully modeled was considered as the reference point, and the weight analysis was based on that day. The treatment lasted for 40 days before the mice were sacrificed. On the day of termination, blood was sampled from mice eyes and euthanize. The weight of the spleen was recorded for further research. Lifted the colon carefully pull until the cecum is visible and measure the length of colon. Thereafter, a part of the colon (about 1 cm) was stored at −80°C until the extraction of RNA and proteins. Another 1 cm of the colon was stored in paraformaldehyde (PFA) for histopathological examination.

### Histopathological Examination

Colon tissues were subjected to the following procedures: routine fixation, decalcification, and paraffin embedding. Subsequently, 4-μm tissue sections were separated and stained with hematoxylin and eosin (H&E).

### Immunohistochemistry

Sections of colon tissues (4 µm) were incubated in Tris buffer (10 mm Tris-HCl, (pH 8.0), 150 mm NaCl), and then incubated overnight with rabbit anti-mouse TNF-α (1:200) at 4°C. Horseradish peroxidase activity was detected using 3,3′-diaminobenzidine and H_2_O_2_. The sections were counterstained with 0.5% methyl green. Quantitative analysis of the immunohistochemistry images was done using Image Pro Plus software.

### Statistical Analysis

All values were expressed as mean ± standard error of the mean (SEM). All statistical analyses were performed using the GraphPad Prism software. Differences between groups were analyzed using the two-tailed Student *t*-test and one-way ANOVA. All statistical analysis was performed using GraphPad Prism Software (version 8.01). Statistical significance was achieved when p < 0.05.

## Results

### Hydrostatin-SN1 Inhibits MAPK Pathway Activation in BMDM Cells

**Figure 1:**
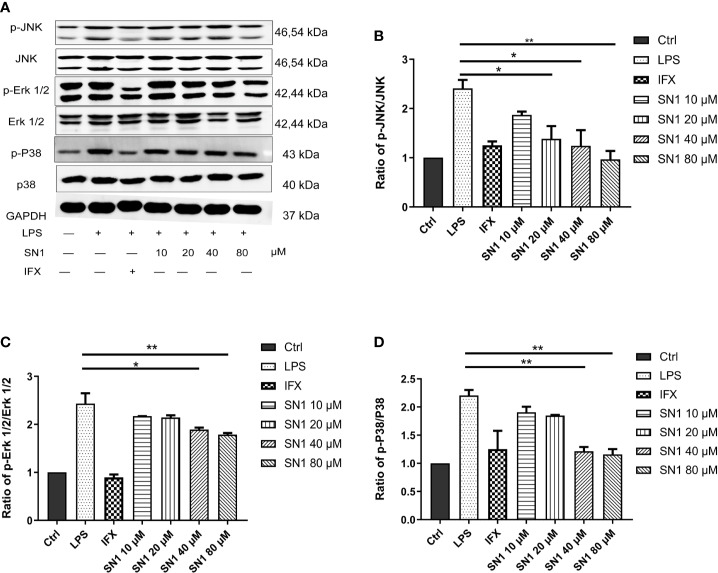
Anti-inflammatory effects of Hydrostatin-SN1 on LPS-induced MAPK pathway activity in BMDM cells. **(A)** Hydrostatin-SN1 inhibited LPS-induced MAPK and NF-κB pathway activation through the phosphorylation of JNK, ERK1/2, and p38. Relative protein levels were quantified using Quantity One software and expressed as optical density ratio. **(B)** Hydrostatin-SN1 decreased the phosphorylation of JNK. **(C)** Phosphorylation of ERK was decreased in Hydrostatin-SN1-treated cells. **(D)** Phosphorylation of p38 was decreased in Hydrostatin-SN1-treated cells. *P < 0.05, **P < 0.01 in Hydrostatin-SN1-treated cells vs. LPS-treated cells.

To investigate the anti-inflammatory effect of Hydrostasin-SN1 *in vitro*, an LPS-induced inflammatory BMDM cell model was developed. We analyzed the MAPK pathway, the key signaling pathway downstream of TNFR1, by western blot (**Figure 1A**). The results showed that LPS dramatically enhanced phosphorylation in BMDM cells, while Hydrostatin-SN1 significantly inhibited the phosphorylation induced by LPS (**Figure 1**). The phosphorylation of JNK (**Figure 1B**), ERK 1/2 (**Figure 1C**), and p38 (**Figure 1D**) were decreased by infliximab and Hydrostatin-SN1.

### Hydrostatin-SN1 Inhibits Expression of Inflammatory Cytokines in LPS-Stimulated BMDM Cells

**Figure 2:**
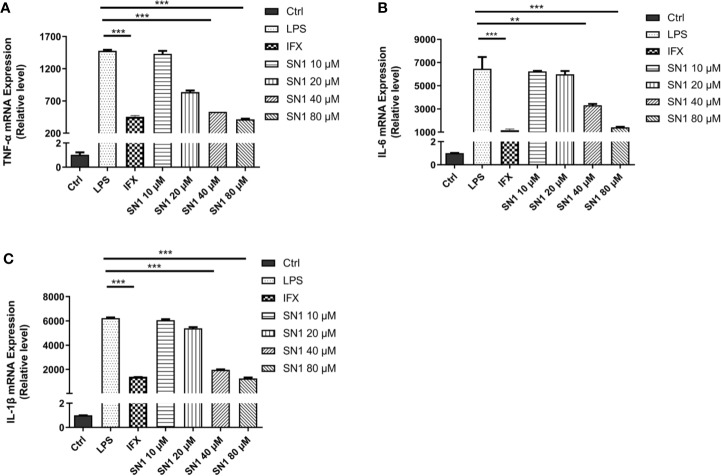
Anti-inflammatory effects of Hydrostatin-SN1 on LPS-induced inflammatory cytokines mRNA expression in BMDM cells. The mRNA expression of TNF-α **(A)**, IL-6 **(B)**, and IL-1β **(C)** was analyzed by RT-PCR at 6 h post LPS stimulation. **P < 0.01, ***P < 0.001 in Hydrostatin-SN1-treated cells vs. LPS-treated cells.

We also performed real-time polymerase chain reaction to detect the expression of inflammatory cytokines. It was observed that Hydrostatin-SN1 with dosage of 80 μm significantly inhibited the expression of TNF-α (*P* < 0.001), IL-6 (*P* < 0.005), and IL-1β (*P* < 0.001) in BMDM cells after 6 h of incubation (**Figures 2A–C**).

### Hydrostatin-SN1 Improves the Survival Rate of LPS-Induced Acute Shock Model Mice

**Figure 3:**
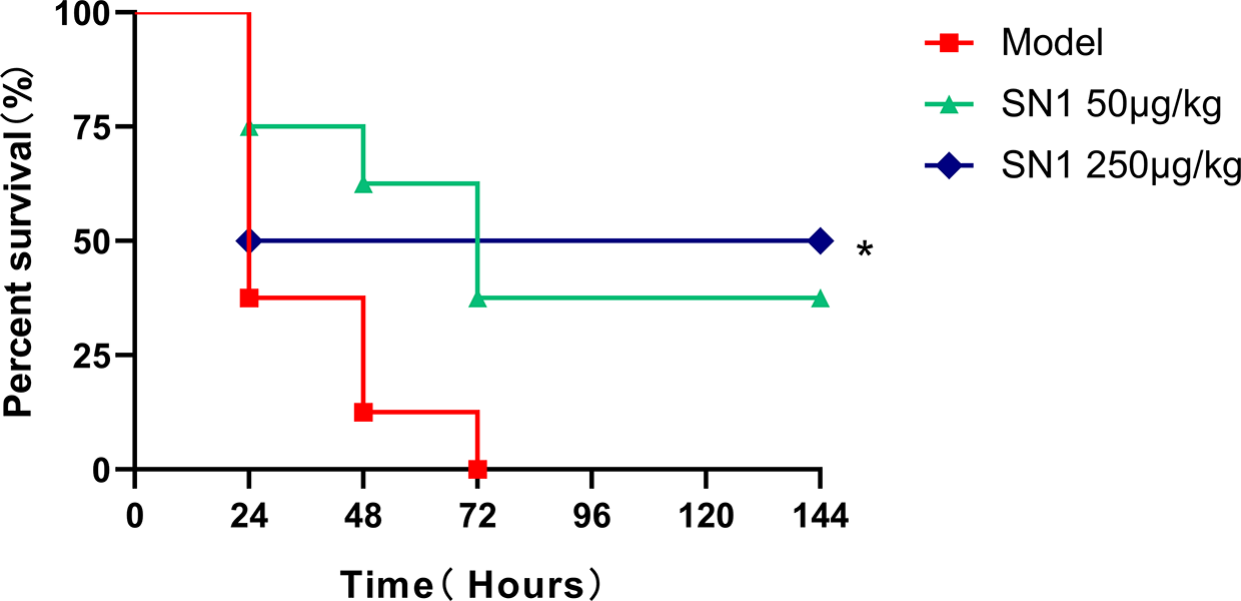
Survival rate of mice injected with LPS and treated with Hydrostatin-SN1. C57BL/6 mice were injected with 15 mg/kg LPS or treated with 50 and 250 μg/kg Hydrostatin-SN1. Mortality was monitored 6 h after injection. Statistical analyses were performed using the log-rank (Mantel–Cox) test. *P < 0.05.

To identify the anti-acute inflammatory effect of Hydrostatin-SN1 *in vivo*, LPS-induced acute shock model was established in C57BL/6 mice (n=8). All the mice in the model group were dead within 72 h after intraperitoneal injection of 15 mg/kg LPS. In contrast, the survival rate of mice in the Hydrostatin-SN1 groups (50 and 250 μg/kg) was significantly better (**Figure 3**).

### Hydrostatin-SN1 Alleviates the Symptoms of Colitis in IL-10 KO Mice

**Figure 4:**
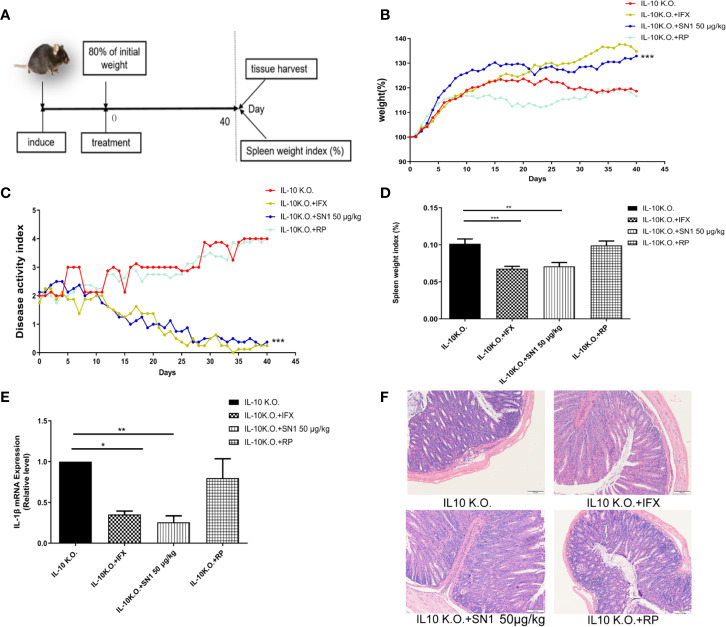
Effects of Hydrostatin-SN1 (dosage of 50 μg/kg) on pathological parameters of colitis in IL-10 knockout mice. **(A)** The workflow shows the design of this experiment. The weights of the mice treated with infliximab or Hydrostatin-SN1 were recorded as the initial weight. **(B)** Greater body weight gain was observed in the IL-10 knockout mice group treated with Hydrostatin-SN1 and infliximab. **(C)** The disease activity index was decreased in the groups of IL-10 knockout mice treated with Hydrostatin-SN1 and infliximab. **(D)** The spleen weight index was calculated for each group, and Hydrostatin-SN1 reduced the spleen weight index in IL-10 knockout mice. **(E)** The mRNA expression of inflammatory cytokines in the colon. mRNA expression levels were determined by real-time PCR, using specific primers for IL-1β. **(F)** Microscopic images of the colons are shown by hematoxylin–eosin staining. Magnification ×200. Values represent the mean ± SEM, *P < 0.05, **P < 0.01, ***P < 0.001 of Hydrostatin-SN1-treated group vs. the IL-10 KO group.

IL-10-KO mice develop Th1 type chronic colitis, which has similar symptoms as Crohn’s disease in humans (Alfen et al., 2018). Thus, we used the IL-10 KO mouse model to assess the anti-inflammatory effect of Hydrostatin-SN1 in IBD. The workflow used for the experiment has been depicted in **Figure 4A**. We first examined the effect of Hydrostatin-SN1 on body weight loss in IL-10 KO mice. As shown in **Figure 4B**, the mice that were administered Hydrostatin-SN1 presented higher weight gain than those in the model group (*P* < 0.001). The disease activity index (DAI) of the mice that were administered Hydrostatin-SN1 decreased significantly (*P* < 0.001), as shown in **Figure 4C**. In addition, the spleen index of the Hydrostatin-SN1-treated mice was significantly reduced compared to that of the IL-10 KO mice (*P* < 0.01) (**Figure 4D**). We also performed RT-PCR to analyze the expression of proinflammatory factor IL-1β in the colon tissue of IL-10 KO mice. The results demonstrated that the expression of IL-1β was inhibited in the colon tissues of Hydrostatin-SN1-treated IL-10 KO mice (*P* < 0.01), unlike that in IL-10 KO mice who were administered saline (**Figure 4E**). Furthermore, histological injury was ameliorated after treatment with Hydrostatin-SN1. The results from the H&E staining showed that the colon of IL-10 KO mice exhibited severe epithelial structure destruction and extensive inflammatory cell infiltration. On the contrary, the colon tissue of Hydrostatin-SN1-treated IL-10 KO mice showed relatively complete epithelial structure and less inflammatory cell infiltration (**Figure 4F**).

### Hydrostatin-SN1 Inhibits Inflammation in IL-10 KO Mice

**Figure 5:**
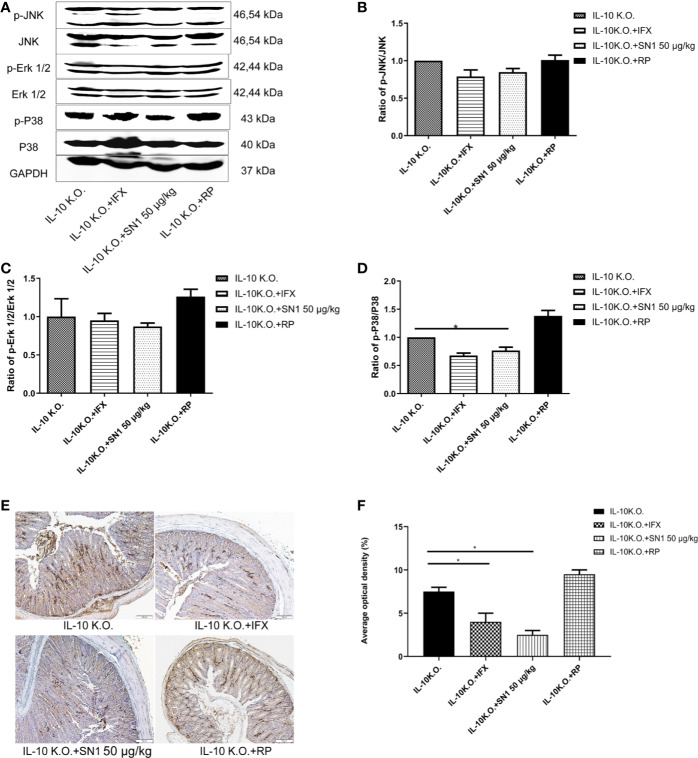
Anti-inflammatory effect of Hydrostatin-SN1 in IL-10 knockout mice. **(A)** Phosphorylation of JNK, ERK1/2, and P38 in each group was analyzed by western blot. Relative protein levels were quantified using Image J software and expressed as optical density ratio. **(B)** Hydrostatin-SN1 decreased the phosphorylation of JNK in IL-10 KO mice. **(C)** The phosphorylation of ERK was decreased in Hydrostatin-SN1 treated mice. **(D)** The phosphorylation of p38 was decreased in Hydrostatin-SN1 treated mice. **(E)** TNF-α expression in the colon tissue in each group. Colon tissues were treated with anti-TNF-α antibody, counterstained with hematoxylin. Magnification ×200. **(F)** Stained areas were calculated by Image Pro plus and the positive expression rates were evaluated. The values represent mean ± SEM, *P < 0.05 for Hydrostatin-SN1 treated group vs. the IL-10 KO group.

We analyzed the level of inflammation in IL-10 KO mice, treated with Hydrostatin-SN1. Western blot was performed to detect the phosphorylation of JNK, ERK1/2, and p38. As shown in **Figure 5**, it was seen that Hydrostatin-SN1 inhibited the phosphorylation of JNK (**Figure 5B**), ERK1/2 (**Figure 5C**), and p38 (**Figure 5D**) in the colon tissue of IL-10 KO mice. In addition, immunohistochemistry was performed to detect the level of TNF-α in the colon of IL-10 KO mice. We found increased TNF-α level in the colon tissue of IL-10 KO mice, which was reversed with Hydrostatin-SN1 treatment (**Figure 5E**). Quantification results of the positive effect area are presented in **Figure 5F**, which showed thatHydrostatin-SN1 decreased the expression of TNF-α in IL-10 KO mice (*P* < 0.05).

## Discussion

In this study, we examined the anti-inflammatory activity of Hydrostatin-SN1 in spontaneous colitis mouse model and in BMDM cells for the first time. In vitro, Hydrostatin-SN1 suppressed TNFR1-mediated mRNA expression of pro-inflammatory cytokines and inhibited the phosphorylation of TNFR1 downstream signaling pathways. In vivo, Hydrostatin-SN1 improved the survival rate of LPS-induced acute shock model mice and ameliorated the clinical manifestations and inflammation-related pathological damage to the colon tissue in IL-10 KO mice. Furthermore, consistent with the *in vitro* experiment results, Hydrostatin-SN1 reduced the production of inflammatory cytokines and inhibited the phosphorylation of TNFR1 downstream signaling pathways in the injured tissues. These data further confirmed that Hydrostatin-SN1 has a broad-spectrum anti-inflammatory activity, and its underlying mechanism is associated with the MAPK pathway and TNF-α. Inflammatory disorders have been associated with diverse diseases, and insights into anti-inflammatory targets will help in developing a variety of novel methods to explore new drugs (Fullerton and Gilroy, 2016).

TNF-α is a classical pro-inflammatory cytokine that has been proven to be an efficacious therapeutic agent in a variety of diseases, such as RA, IBD, and psoriasis (Annibaldi and Meier, 2018). It has been demonstrated that TNF-α activates pro-inflammation factors, programmed-cell death pathway, and tissue injury *via* TNFR1 (Bradley, 2008), and thus TNFR1 inhibitors have become a popular target for clinical treatment of inflammatory diseases. In clinical application, five anti-TNF-α agents have been approved by the US Food and Drug Administration, including monoclonal antibodies (infliximab, adalimumab, certolizumab, and golimumab) and solubility TNF receptor (etanercept), used to treat inflammatory diseases associated with TNFR1 (Lis et al., 2014; Sedger and McDermott, 2014; Kalliolias and Ivashkiv, 2016; Li et al., 2017). Despite their therapeutic effects, these drugs block TNF, resulting in several negative side effects, including low rate of disease remission, development of fatal adverse effects such as lupus-like symptoms and lymphoma, and production of antibodies against biological TNF inhibitor (Feldmann and Maini, 2015; Kalliolias and Ivashkiv, 2016). In addition, antibodies are expensive and often do not cross the blood–brain barrier, and may cause injection site reactions or infusion reactions (Schabert et al., 2013; Kaltsonoudis et al., 2014; Sedger and McDermott, 2014). Thus, the development of small molecule compounds or peptides, which specifically inhibit the biological function of TNFR1, has a great potential in the treatment of inflammatory diseases associated with TNFR1.

Mitogen-activated protein kinase (MAPK) and nuclear factor-κB (NF-κB) signal pathway had been improved to be activated by Toll-like receptors (TLRs) regulating immune responses (Li et al., 2014). Depletion of IL-10 critically inhibit the anti-inflammatory activity of p38 (Raza et al., 2017). Mice with inhibition of MAPK signal pathway presented a downregulation the expression of IL-1β in acute injury in intestinal ischemia reperfusion model (Zheng D.-Y. et al., 2016). In this research, we detected the activity of MAPK and NF-κB pathway *via* the phosphorylation of JNK, ERK, and p38. We have also detected the mRNA expression of IL-1β in mice. The results suggested that Hydrostatin-SN1 regulate the activity of NF-κB pathway.

As a TNFR1 antagonist peptide, we believe that Hydrostatin-SN1 might be a potential candidate drug for IBD inflammatory diseases associated with TNFR1. Hence, further studies are required to prove that Hydrostatin-SN1 is highly selective of TNFR1 and exerts anti-inflammatory effects *via* the MAPK pathway.

## Data Availability Statement

All datasets generated for this study are included in the article/supplementary material.

## Ethics Statement

The animal study was reviewed and approved by The Animal Care and Use Committee of the Second Military Medical University.

## Author Contributions

YL, XM, and SW conceived and designed the research. CZ, SG, and JW performed the *in vivo* experiments. AL and KS performed the *in vitro* experiments. CZ, JL, and LQ analyzed the data. YL, XM, and SW contributed reagents/materials/analysis tools. CZ, SG, and JW wrote the paper. All authors contributed to the article and approved the submitted version.

## Funding

This work was supported by The National Key Research and Development Program of China (2018YFC0310900), The National Natural Science Foundation of China (No. 81773627, No. 81274162, and No. 81601682), National Major Scientific and Technological Special Projects for “Significant New Drugs Innovation and Development” (No. 2019ZX09301119), and Shanghai Science and Technology Innovation Action Plan (No. 16431904400).

## Conflict of Interest

The authors declare that the research was conducted in the absence of any commercial or financial relationships that could be construed as a potential conflict of interest.
